# Adaptive median filter salt and pepper noise suppression approach for common path coherent dispersion spectrometer

**DOI:** 10.1038/s41598-024-66649-y

**Published:** 2024-07-29

**Authors:** Shouxin Guan, Bin Liu, Shasha Chen, Yinhua Wu, Feicheng Wang, Xuebin Liu, Ruyi Wei

**Affiliations:** 1 Key Laboratory of Spectral Imaging Technology CAS, Xi’an Institute of Optics and Precision Mechanic of Chinese Academy of Sciences, University of Chinese Academy of Sciences, Xi’an, 710119 China; 2 University of Chinese Academy of Sciences, Beijing, 100049 China; 3 School of Optoelectronics Engineering, Xi’an Technological University, Xi’an, 710021 China; 4 Hubei Luojia Laboratory, Wuhan, 430072 China; 5 Wuhan Institue of Quantum Technology, Wuhan, 430072 China; 6 School of Electronic Information, Wuhan University, Wuhan, 430072 China

**Keywords:** Coherent-dispersion, Radial velocity, Salt-and-pepper noise, Adaptive median filtering, Astronomy and astrophysics, Techniques and instrumentation, Astronomy and astrophysics, Applied optics, Optical techniques, Computer science

## Abstract

The Common-path Coherent-dispersion Spectrometer (CODES), an exoplanet detection instrument, executes high-precision Radial Velocity (RV) inversions by recording the phase shifts of interference fringes. Salt-and-pepper noise caused by factors such as improper operation of the CCD probe/analog-to-digital converter and strong dark currents may interfere with the phase information of the fringe. This lowers the quality of the interfering fringe image and significantly interferes with the RV’s inversion. In this study, an adaptive median filtering algorithm (CODESmF) based on submaximum and subminimum values is designed to eliminate the interference fringe image's salt-and-pepper noise as well as to reduce RV error. This allows the interference fringe image's phase information to be retained more completely. The algorithm consists of two major modules. Pixel Sub-extreme-based Filtered Noise Monitoring Module: discriminates signal pixels and noise pixels based on the submaximum and subminimum values of the pixels in the filtering window. Adaptive Median Filter Noise Suppression Module: the signal pixel is kept at the original value output, the noise pixel serves as the filtering window's center pixel, and the adaptive median filtering procedure is repeated numerous times with various filtering window sizes. According to the experimental findings, the CODESmF outperforms comparable algorithms and works better at recovering interference fringes. More than 90% of the phase/RV error caused by salt-and-pepper noise is typically eliminated by the CODESmF algorithm, and in certain circumstances, it can even remove roughly 98% of the phase error.

## Introduction

The first Jupiter-mass class planet, 51 Pegasi b, was found in 1995 by Mayor and Queloz of the Exoplanet Exploration Group (EEG) in Geneva, Switzerland, close to a Sun-like star (51 Pegasus)^1^, launching the human search for extrasolar planets. For the Earth, distant stars and orbiting planets are close to each other, and stars are typically 1 billion times brighter than Earth-like planets in the visible wavelength band, so the indirect detection methods of Astrometry^2^, Radial Velocity^3^, Transit^4^, Circumstellar disks^5^, Pulsar timing^6^, and Gravitational microlensing^7^ are the most widely used search techniques internationally. The Radial Velocity approach, also known as the Doppler effect method, identifies exoplanets by observing the effect (red-shift or blue-shift) of a star's spectrum. There must be at least one planet orbiting a star if its radial velocity fluctuates according to a sinusoidal law.

Dispersed Fixed Delay Interferometry (DFDI)^8–10^, a novel technology that combines a Michelson interferometer with a spectrograph, was the foundation upon which Erskine and Jian Ge created the Extroplanet Tracker in 2002. The Extroplanet Tracker has been successfully tested on the 2.1 m telescope of Kitt Peak National Observatory (KPNO), USA.

Ruyi Wei^11,12^ developed a Common-path Coherent-dispersion Spectrometer (CODES) for exoplanet detection, produced a laboratory Doppler modeling and demonstration platform, and substituted an asymmetric common-path Sagnac interferometer for the conventional Michelson interferometer. The input light is loaded with interference information when it enters the Sagnac interferometer^13^^,^^14^, and then the light is scattered into two-dimensional interference fringes by the spectrometer before being detected by the CCD detector as a digital signal that can be processed.

During the acquisition of interference fringe images, a variety of factors lead to a large amount of random noise interference in the acquisition and transmission of the image signal, including instrumental noise, photon noise, background light or cosmic ray contamination, noise caused by intrinsic stellar photospheric activity, wavelength calibration errors and errors caused by CCD inhomogeneities^15–17^. The interference fringe image quality is reduced by these random noises, making the RV accuracy decrease dramatically. The noise can be classified according to its probability distribution as Gaussian noise^18^, gamma noise, salt-and-pepper noise^19^, Poisson noise, etc. Among them, random noise disturbances such as improper operation of CCD probes/analog-to-digital converters and the formation of hot pixels on pixels by strong dark currents can be regarded as salt-and-pepper noise^20^. It is a haphazard appearance of dark (pepper) or bright (salt) dots with either black pixels in bright parts or white pixels in dark areas. Since the high intensity and random distribution of the salt-and-pepper noise, when the salt-and-pepper noise appears on the interference fringe image, it will have a significant impact on the interference fringe image quality and RV inversion.

Presently, salt-and-pepper noise is eliminated using techniques like regularization, filtering, and machine learning. An adaptive TV-L1 (ATV1) denoising technique was presented by Thanh et al.^21,22^. This method includes adaptive regularization parameter estimation and can effectively handle images with high-density noise corruption. To identify patterns and similarities in the images that aid in the restoration process, support vector machines^23^ and convolutional neural networks^24^ are employed as learning tools. Literature^25^ uses a blind denoiser based on generative adversarial networks and convolutional neural networks. The most popular and extensively used filtering technique for eliminating impulsive random noise interference is median filtering, which is based on sorting theory. It is most straightforward and efficient. Examples include Adaptive Median Filter (AMF)^26^, Progressive Switching Median Filter (PSMF)^27^, Modified Decision-Based Unsymmetric Trimmed Median Filter (MDBUFLOW)^28^, Based on Pixel Density Filter (BPDF)^29^, Noise adaptive fuzzy switching median filter (NAAF)^30^, and, switching median filter (NAFSMF)^30^.

The median filtering technique eliminates noise by substituting the singular value with the median value, which effectively filters out salt-and-pepper noise while preserving the edge and detail information of the image. The standard median filtering (SMF) algorithm^31^ effectively smooths out background noise with a nonlinear filtering fashion but hard to discriminate between noise and signal points, which frequently causes blurring of image details. Numerous enhanced algorithms are suggested for this aim. The adaptive median filtering (AMF) algorithm^32^ dynamically adjusts the filtering window size based on a predefined window size threshold, which improves the filtering effect on high-density noise. The algorithm uses a fixed window size threshold, which is unable to adaptively adjust the threshold for images with different noise densities, and increasing the window size one at a time is not only time-consuming but also causes image blurring. A selective adaptive median filter (SAMF) is suggested in the literature^33^. This filter first identifies the noisy pixels before dynamically adjusting the filter window size following the constraints until a suitable median is obtained to replace the noisy pixels. However, the replacement values produced by this technique when utilizing a large-size window are not only labor-intensive but also inefficient at replicating the original pixel information. Based on this, literature^34^ suggests a median filter (MDBMF) based on improved decision-making, which processes only noisy pixels and judiciously increases the filter window size. This filter can greatly enhance the performance of high-density noise denoising and operate more quickly. Nevertheless, the method performs the step of keeping the original noise pixels for localized high-density noise, which has an impact on the system's denoising performance. WAN Fengfeng et al.^35^ proposed an adaptive fuzzy median filtering algorithm, using the maximum-minimum method to detect noise, the fuzzy membership function is defined by the mean of the absolute gray difference between the suspected pixels and its neighborhood pixels which has been processed, and the suspected pixels are fuzzy classified by this function. XU Guangyu^36^ proposed an Efficient Switching Median Filter. The pixel points are classified into signal and possible noise points using the two extreme values of the gray value of the image. Polar discard filtering and recursive filtering are performed using a filter window size of 3 × 3 to remove noise and maintain image details. The adaptive feedback median filter^37^ detects corrupted or noisy pixels by decisively analyzing the neighbors. It predicts a local threshold by analyzing the neighbors to decide the adaptive nature of the feedback median filter.

In this study, an adaptive median filtering algorithm (CODESmF) based on the noise detector for submaximum and subminimum values is proposed, which consists of two basic steps, i.e., the noisy pixel points are identified using the submaximum and subminimum values noise detector, and the marked pixels are then repaired using adaptive median filtering. The modeling of salt-and-pepper noise is reviewed in Section “Salt-and-pepper noise model”. The fundamentals of the adaptive median filtering algorithm based on the sub-extreme-based salt-and-pepper noise detector are discussed in Section “Algorithmic principles of CODESmF”. The algorithm’s experiments to handle interference fringe images are described in Section “Experimental results and analysis’’, along with assessment metrics based on RV/phase offset. The summary and conclusion are provided in Section “Summary”.

## Salt-and-pepper noise model

The term “salt-and-pepper noise,” also called “bipolar impulse noise,” refers to a type of bright and dark spot noise that is typically produced by image sensors, transmission channels, decoding procedures, etc. The image appears to have salt and pepper sprinkled haphazardly throughout (black negative noise points correspond to pepper, while the white positive noise points correspond to salt)^38^. The salt-and-pepper noise in the interference fringe image only affects a small portion of the pixels, the locations of the noise points are randomly distributed, and both positive and negative noise points are equally likely to exist. The amplitude of salt-and-pepper noise is nearly the same, it frequently corresponds to the maximum or minimum of the gray level, and the gray value of a pixel polluted by pepper noise has no bearing on the gray value of an uncontaminated pixel nearby.

Let I represent the original, noise-free interference fringe image with M × N in size. Then I (i,j) denote the gray value of the pixel with coordinates at position (i,j) in the interference fringe image, where $(i,j)\in A\equiv \{1,\cdots ,M\}\times \{1,\cdots ,N$. Let [S_min_, S_max_] represent the interval of gray values of all pixels in the interference fringe image I, so $I \left(\text{i},\text{j}\right)\in [\text{Smin},\text{Smax}]$. The original gray value of the pixel point will be changed to S_min_ or S_max_ when the interference fringe image is tainted with salt-and-pepper noise. Therefore, defining the interference fringe image contaminated by salt-and-pepper noise as A, the gray value of its pixel position as (i,j) is:

1 $$A\left(\text{i},\text{j}\right)=\left\{\begin{array}{lll}{S}_{min},&  {\text{with probability}} & p \\ {S}_{max},&  {\text{with probability}}& q\\ I(i,j), &  {\text{with probability}} & 1-p-q\end{array}\right.,$$

where I(i,j) is the salt-and-pepper noise-free pixel point’s gray value, p + q is the noise density, and S_min_ and S_max_ have the same probability, i.e., p = q. The probability distribution formula for salt-and-pepper noise is as follows, where Z denotes the gray value. Many times, saturation values a and b are taken to be the highest and lowest values that can be used in digital photographs. For an 8-bit image, this means that a = 0 (black) and b = 255 (white).

2 $$P(z)=\left\{\begin{array}{cc}Pa& Z=a\\ Pb& Z=b\\ 0& \text{ otherwise}\end{array}\right.$$

The CODES offers better energy efficiency and detection accuracy, while it also has more challenging data processing duties. Instead of measuring the wavelength drift of the stellar spectrum as is done by traditional radial velocity measurement instruments, the CODES converts the spectral lines of stellar light into interference fringes and calculates the RV by measuring the phase change of the interference fringes^39^. Three dimensions of information are present in two-dimensional interference fringes captured by a CCD: the interference dimension (direction) and the dispersion dimension (direction) on the target surface of the CCD detector, as well as the optical energy dimension represented by the light and dark variations of the interference fringes. Interference fringe images contain interference information in the slit direction, spectral information along the dispersion direction, and bright and dark variations of the fringes that capture information about light energy. The interference fringe’s profile changes when either of its dimensions changes and this change in profile alters the interference fringe’s phase information, making CODES more sensitive to external factors. Numerous instrument effects and ambient changes can be confused for velocity variations due to the equipment’s exceptionally high sensitivity. The target signal travels through the telescope system and CODES before forming two-dimensional interference fringes on the CCD detector. The interference fringes’ morphology is somewhat influenced by the mirror system, the focal plane system, the spectrometer system, the CCD camera, and other factors. Changes in the interference fringes might result from minor disturbances and show up as RV variations in the target star. S_min_ or S_max_ will take the place of the pixel point's original gray value when the interference fringe image has been contaminated with salt-and-pepper noise. The information carried by the interference fringes is severely disrupted when salt-and-pepper noise shows up on them. This is manifested by a large error in the RV inversion.

## Algorithmic principles of CODESmF

For salt-and-pepper noise, it is frequently the case that not all pixel points need to be processed and given values. When normal pixel points in an interference fringe image are filtered, the normal pixel points’ pixel values are altered, destroying the original clean pixels and seriously distorting the image.

A switching median filtering processing strategy is suggested as a result: firstly, all pixels are classified into noise and signal pixels according to specific discriminative criteria; then, noise and signal pixels are processed individually, noise pixels are replaced with the neighborhood median value by spatial correlation, while signal pixels remaining with their original value. Processing success depends on the selection of the discrimination-determining criteria.

**Figure 1:**
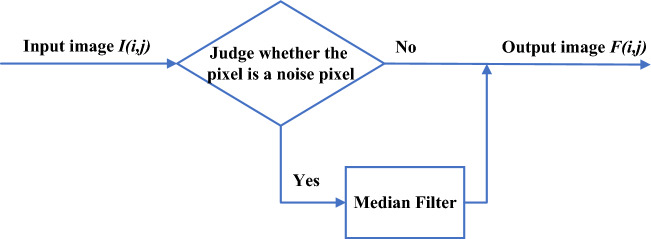
Switching median filtering algorithm schematic diagram.

As shown in Fig. 1 is a schematic diagram of the switched median filtering algorithm, where I (x,y) is the gray value of the center pixel point, then the output of the switched median filtering F (x,y) can be expressed by the following equation:

3 $$F\left(x,y\right)=\left\{\begin{array}{c}I(x,y),\hspace{0.25em}\hspace{0.25em}\hspace{0.25em}\hspace{0.25em}|I(x,y)-M(x,y)|<T\\ M(x,y),\hspace{0.25em}\hspace{0.25em}\hspace{0.25em}\hspace{0.25em}|I(x,y)-M(x,y)|\ge T\end{array}\right.,$$

where M (x,y) is the median value of each pixel point within the current window, and T is a pre-determined threshold.

### Image preprocessing

The filter window size of the median filtering algorithm can significantly affect the performance of the algorithm. For instance, it is important to utilize a bigger window when dealing with high-density noise, yet at this point, the median filtering algorithm struggles to preserve image details. The median filtering method has a drawback when the amount of salt-and-pepper noise is high: If the filtering window is too small and there are too many noisy pixels present, the gray value of the center pixel determined by the median filtering technique will be impacted by the noisy pixels.

By efficiently addressing the issues that median filtering methods have while dealing with high-density noise, dynamic windows can enhance the algorithms’ performance^40^. The so-called dynamic window is used to design the filter window size of the median filtering algorithm, starting from a size of 3 × 3 and gradually increasing the size until the adaptive condition is satisfied or the size reaches a limit value^41^. Adaptive conditions can be selected according to the environment in which the median filtering algorithm is used.

**Figure 2:**
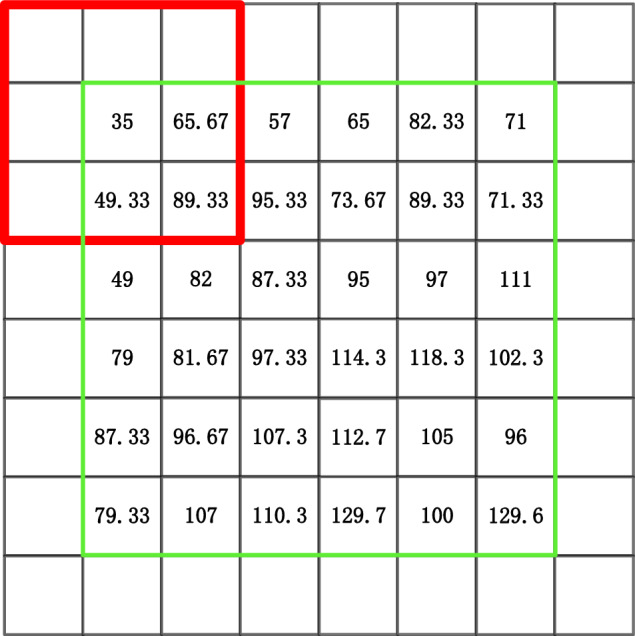
Schematic of the filtering operation at the image boundary with a window size of 3 × 3.

It is also required to find a solution to the issue that some boundary pixel locations cannot generate filter windows after choosing the dynamic window approach. As demonstrated in Fig. 2, when a filter window of 3 × 3 size is chosen, the noisy pixel points at the image boundary cannot be processed because the window’s center pixel is a pixel point at the image boundary and the window’s coverage area is not filled with pixel points.

To address the above problems, this algorithm expands the boundaries of the image using a method based on mirror copying of the image boundaries^42^. Fold the R pixels near the image boundary outward, i.e., mirror copy the R pixels outward with the image boundary as the axis of symmetry. R stands for the maximum filter window’s edge-centroid distance. The original image region is cropped to produce the filtered output image after filtering is complete. By using this technique, it is made sure that the pixel points at the edge of the image are filtered and that the expanded region retains the same grayscale characteristics as the edge of the image.

Equation (4) shows the corresponding grayscale matrix of an image, and the expanded image matrix (5) can be obtained by mirroring and copying 3 pixels outward for expansion.

4 $$\left[\begin{array}{ccc}36& 65.67& 57\\ 49.33& 89.33& 95.33\\ 49& 82& 87.33\end{array}\right]$$

5 $$\left[\begin{array}{ccccccccc}87.33& 82& 49& 49& 82& 87.33& 87.33& 82& 49\\ 95.33& 89.33& 49.33& 49.33& 89.33& 95.33& 95.33& 89.33& 49.33\\ 57& 65.67& 36& 36& 65.67& 57& 57& 65.67& 36\\ 57& 65.67& 36& 36& 65.67& 57& 57& 65.67& 36\\ 95.33& 89.33& 49.33& 49.33& 89.33& 95.33& 95.33& 89.33& 49.33\\ 87.33& 82& 49& 49& 82& 87.33& 87.33& 82& 49\\ 87.33& 82& 49& 49& 82& 87.33& 87.33& 82& 49\\ 95.33& 89.33& 49.33& 49.33& 89.33& 95.33& 95.33& 89.33& 49.33\\ 57& 65.67& 36& 36& 65.67& 57& 57& 65.67& 36\end{array}\right]$$

### Salt-and-pepper noise detection based on submaximum and subminimum values

The threshold value T is used by conventional switching median filtering methods to assess whether a pixel point is contaminated or not. If the threshold T is set too high, certain salt-and-pepper noise points may be mistaken for non-salt-and-pepper noise points, making the processing impact less noticeable; similar to the above, if the threshold T is set too low, certain non-salt-and-pepper noise points may be mistaken for salt-and-pepper noise points, resulting in distortion in the processed image.

**Figure 3:**
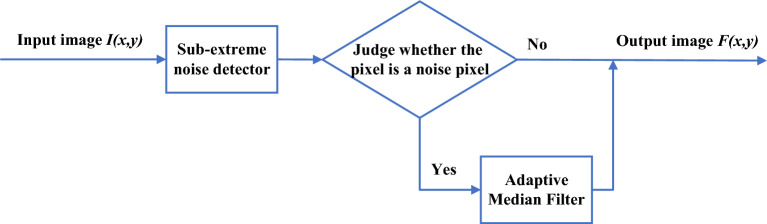
Schematic diagram of the sub-extreme-based adaptive median filtering algorithm.

This algorithm evaluates all of the pixel points using the submaximum and subminimum values salt-and-pepper noise detector, as illustrated in Fig. 3, and the salt-and-pepper noise threshold range is defined by the next-largest and next-smallest pixel points surrounding the center pixel. The adaptive median filtering algorithm is used to process salt-and-pepper noise points outside the threshold range, while the original gray value is maintained for non-salt-and-pepper noise points inside the threshold range.

It is significant to note that the decision to use sub-extreme values rather than extreme values for detecting salt-and-pepper noise is due to the presence of emission/absorption peaks with larger/smaller gray values in the interference fringe image, which may be present in the form of extreme values within the filtering window. The extreme median filtering algorithm will then mistakenly categorize these pixel points as salt-and-pepper noise points, resulting in a loss of certain details in the interference fringe image and a disruption of the RV inversion.

Define the filter window W_2r + 1_ (i,j), which is centered on the pixel point (i,j) and has the size (2r + 1) (2r + 1), W _2r + 1_ (i,j) can be expressed as

6 $$\begin{array}{c}{W}_{2r+1}(i,j)=\{I(i+m,j+n)\},\\ m,n\in \{-r,\cdots ,0,\cdots ,r\},\end{array}$$

where r is a positive integer and I (i, j) denote the gray value of the pixel point (i,j).

Assuming that r = 1 and (x, y) is the filter window’s center points, making W_3_ (i,j) is a 3 × 3 sliding filter window centered on (x,y), eight-pixel points in the neighborhood of the center point (x,y) can be obtained as shown in Eq. (7), which in turn produces the second-largest value of the grayscale values in the filter window, f_smax_ (x,y), and the second-smallest value of the grayscale values, f_smin_ (x,y), as given in Eq. (8).

7 $${W}_{3}(i,j)={W}_{3}(x,y)=\left[\begin{array}{ccc}I(x-1,y-1)& I(x-1,y)& I(x-1,y+1)\\ I(x,y-1)& I(x,y)& I(x,y+1)\\ I(x+1,y-1)& I(x+1,y)& I(x+1,y+1)\end{array}\right]$$

8 $$\begin{array}{c}{f}_{smax}(x,y)=sec\,max\{f(i,j)\}\\ {f}_{smin}(x,y)=sec\,min\{f(i,j)\}\end{array}$$

In Eq. (9), it is demonstrated that the range of salt-and-pepper noise judgment thresholds [α, β] can be calculated as the range of salt-and-pepper noise judgments within the window W_2r + 1_ (i,j) based on f_smax_ (x, y) and f_smin_ (x,y), where γ is taken as an empirical value, γ = 0.25 ref^43^.

9 $$\left\{\begin{array}{c}\alpha ={f}_{smin}(x,y)-\gamma \cdot \left[{f}_{smax}(x,y)-{f}_{smin}(x,y)\right]\\ \beta ={f}_{smax}(x,y)+\gamma \cdot \left[{f}_{smax}(x,y)-{f}_{smin}(x,y)\right]\end{array},0<\gamma \le 1\right.$$

The pixel point at the window's center is identified as a non-salt-and-pepper noise point if its gray value, f (x,y), is within the range of [α,β]. If f (x,y) falls outside the threshold range [α, β], the pixel point is identified to be a salt-and-pepper noise point.

10 $$I(i,j)\in \left\{\begin{array}{l}N\hspace{0.25em}\hspace{0.25em}\hspace{0.25em}\hspace{0.25em}I(i,j)<\alpha \, {\text{o}}{\text{r}} \, I(i,j)>\beta \\ S\hspace{0.25em}\hspace{0.25em}\hspace{0.25em}\hspace{0.25em}\alpha <I(i,j)<\beta \end{array},\right.$$

where N stands for the collection of pixels affected by noise, and S stands for the collection of pixels unaffected by noise. If $I (\text{i},\text{j})\in \text{N}$, it is a noisy pixel, and if $I (\text{i},\text{j})\in \text{S}$, it is a signal pixel then.

A noise identification matrix K of equal size as the noise-containing image is defined to identify the noisy pixels. Each pixel in the noise-containing image is detected point-by-point using the procedure described in Eq. (8). If $I (\text{i},\text{j})\in \text{N}$, the corresponding position in matrix K is labeled 1, and if $I (\text{i},\text{j})\in \text{S}$ , it is labeled as 0.

11 $$K=\left\{\begin{array}{ll}{K}_{0} &  I(i,j)\in S \\ {K}_{1}& I(i,j)\in N\end{array}\right.$$

### Adaptive median filtering

The interference fringe image A with noise contamination is subjected to mirror copying of the image boundary. With the image boundary as the axis of symmetry, R = 9 pixels are mirror copied outward to obtain the interference fringe noise image $\widetilde{\text{A}}$ after expanding the boundary. Each pixel of the image $\widetilde{\text{A}}$ is detected point by point, and it is determined whether it belongs to a noise pixel or not by using Eq. (10), thus obtaining the noise identification matrix K.

The pixel $\widetilde{\text{I }(\text{i},\text{j})}$ that corresponds to K = K_1_ in image $\widetilde{\text{A}}$ is used as the center pixel for a median filtering operation. The grayscale values of each pixel point in the filtering window W_2r + 1_ (i,j) are sorted to create the sequence W _sort_ (i,j), and the grayscale value of $\widetilde{\text{I }(\text{i},\text{j})}$ is replaced with the median value of the sequence W _sort_ (i,j), M(x, y).

12 $$M(x,y)=\text{median}\left\{{W}_{\text{sort }}(i,j)\right\},K={K}_{1}$$

**Figure 4:**
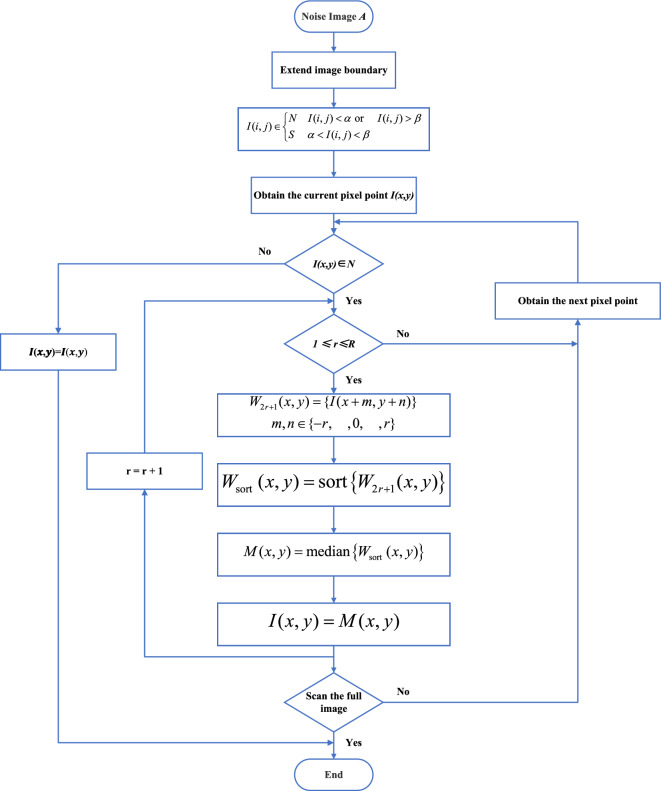
Algorithm flow schematic for the adaptive median filtering method using sub-extreme values.

In this procedure, the filter window size W_2r + 1_ (i,j) is gradually increased from 3 × 3 to 19 × 19, and the median filtering operation is repeatedly carried out. Fig 4 depicts the algorithm’s flow.

## Experimental results and analysis

### Evaluation criteria based on RV/Phase offset

The Doppler effect^44,45^ is caused by a shift in the radial component of the star's orbital speed along the direction of the Earth as it moves in a tiny circular orbit under the influence of the planet’s gravitational pull. By detecting the periodic shift in the spectral Doppler induced by the planet's traction to the star, the RV approach infers the existence of planets indirectly.

The wavelength calculation formula for the Doppler effect is

13 $$\uplambda ={\uplambda }_{0}+\Delta\uplambda \approx {\uplambda }_{0}\left(1+\frac{v}{c}\right)$$

Among them, λ is the wavelength of the stellar spectral line received by the observer, λ_0_ is the spectral line wavelength when the star radial velocity is 0, Δλ is the wavelength offset of the stellar spectral line, c is the speed of light 299792458 m/s, ν is the radial velocity of the star relative to the observer, and the movement away from the observer is positive.

Eq. (14) can be calculated by (13). In (14), the wavelength is converted into a wavenumber to represent, then (15) and (16) can be derived, where k represents the wavenumber of the stellar spectral line that the observer received, k_0_ is the spectral line wavenumber when the star radial velocity is 0, Δk is the wavenumber offset of the stellar spectral line.

14 $$\frac{\Delta\uplambda }{{\uplambda }_{0}}=\frac{v}{c}$$

15 $$\frac{\Delta\uplambda }{{\uplambda }_{0}}=\frac{\uplambda -{\uplambda }_{0}}{{\uplambda }_{0}}=\frac{1/k-1/{k}_{0}}{1/{k}_{0}}=\frac{{k}_{0}-k}{k}=-\frac{\Delta k}{k}$$

16 $$\frac{\Delta k}{k}=-\frac{v}{c}$$

For a light source with an amplitude of I_0_ and a wavenumber of k, the following formula can be used to determine the intensity of the interference fringes produced at the optical path difference (OPD) d:

17 $$I={I}_{0}\left[1+\text{cos}(2\uppi dk)\right]$$

The OPD and the wave number k of the light source, as stated in (17), define the phase of the interference fringes. As a result, (18) shows the phase difference at the same OPD before and after the Doppler frequency shift.

18 $$\Delta\upphi =2\uppi d(k-{k}_{0})=2\uppi d\Delta k$$

Substituting (16) into (18), the corresponding relationship between radial velocity ν and phase difference Δφ can be obtained, as shown in (19) and (20).

19 $$\Delta\upphi =-2\uppi d k v / c$$

20 $$v=-\frac{c}{2\uppi dk}\Delta\upphi =-\frac{c\uplambda }{2\uppi d}\Delta \phi $$

That is, after obtaining the phase difference Δφ of the interference fringes before and after the stellar Doppler frequency shift through CODES measurement, the corresponding stellar radial velocity ν can be calculated according to (20).

For the interference fringe image A with noise contamination, the salt-and-pepper noise interferes with the phase information carried by the interference fringes, resulting in an error in the phase difference $\overline{\Delta \phi  }$ of the interference fringes that CODES eventually collected. As a result, the RV produced from the inversion is eventually biased.

21 $$\overline{v }=-\frac{c}{2\uppi dk}\overline{\Delta\upphi  }=-\frac{c\uplambda }{2\uppi d}\overline{\Delta\upphi  }$$

Therefore, utilizing the efficiency of correcting the RV/phase offset as a criterion for algorithm evaluation, this work examines the denoising effect of the algorithm as well as the recovery of the interference fringe image.

### Experimental data acquisition

The laser interference fringes obtained from the CODES principle verification device^11^ are selected as the experimental objects to assess the effectiveness of the proposed algorithm.

**Figure 5:**
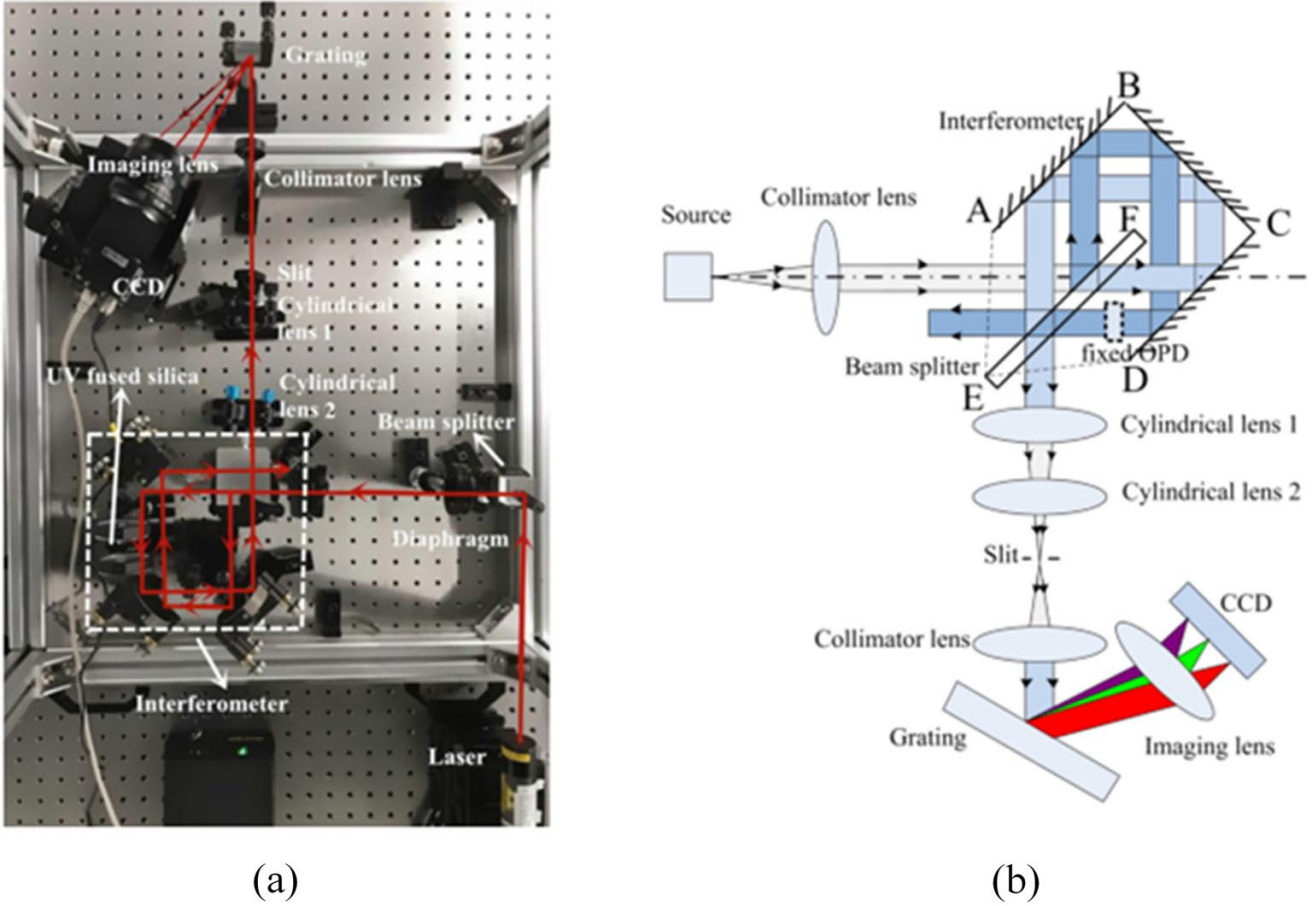
(**a**) The set-up of CODES; (**b**) Schematic diagram of CODES.

The experimental CODES device was constructed in a clean, temperature-controlled laboratory. Fig 5 depicts the optical principle and the device’s related arrangement, and Table 1 lists the specifications of the major optical parts. A He–Ne laser was selected as the narrowband light source. The interferometer subsystem consists of an unpolarized cube beam splitter, three mirrors, and four UV-fused silica cubes with a refractive index of 1.457 and a total length of 34 mm. Silica cubes produced a fixed OPD of 15.538 mm. The period of the interference fringe is adjusted by the pitch angle of the reflector AB.

**Table 1:** The parameters of the main optical elements.

Parameter	Value
Focal length of cylindrical lens 1 (mm)	150
Focal length of cylindrical lens 2 (mm)	50
Slit width (μm)	40
Focal length of the collimator lens (mm)	125
Grating (1/mm)	1200
Focal length of the imaging lens (mm)	85
CCD detector	2592*1944 with 6 μm pixels

**Figure 6:**
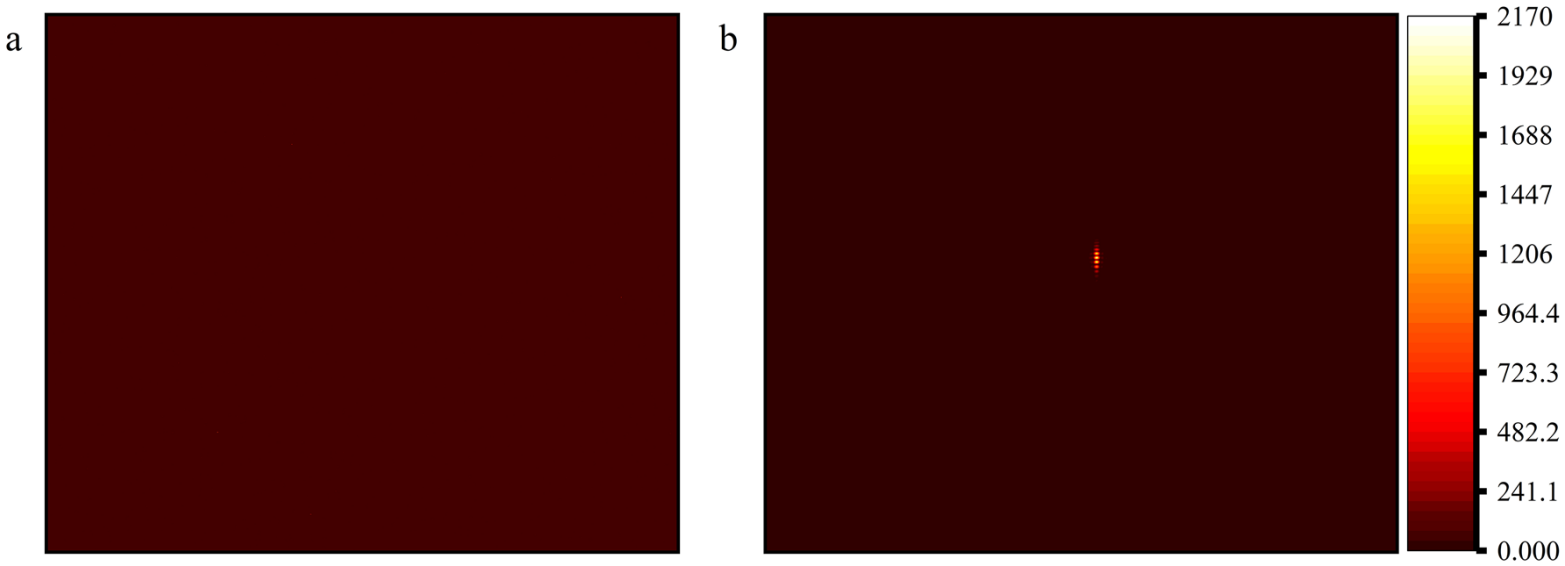
Base image and interference fringe image. (**a**) the base image; (**b**) the overall image after de-base.

The raw interference requires the following processing to extract the radial velocity data from the obtained interference fringes. The first is to subtract the base, whereby bias voltages cause the CCD to emit various numbers of electrons even in the absence of exposure, which must be eliminated while processing the interference fringe image. Specifically, the initial interferogram removes the background image that would exist if no light entered the coherent dispersion system. Figure 6 displays the first set of interference fringe images used as a test subject (every presentation after this one is based on the first set of interference fringe images).

**Figure 7:**
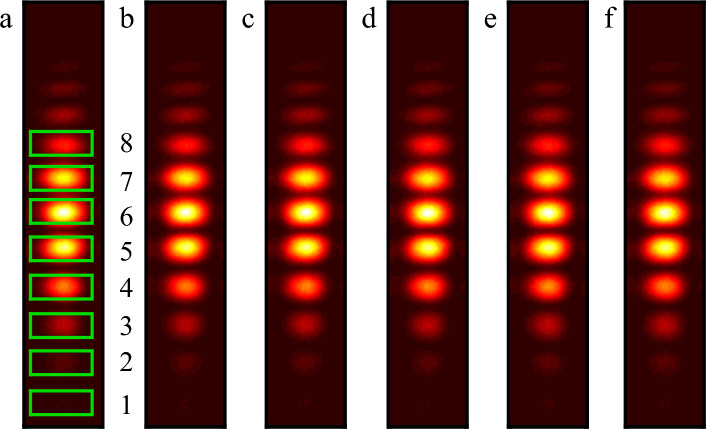
Six sets of interference fringe images as the processing object. Eight interference fringe spots with varying brightness are identified as spots 1 through 8 in the green box.

The interference fringe images captured at six successive exposure periods were chosen as processing objects under identical experimental conditions. The interference brought on by changes in the surroundings on the images of different interference fringes can be disregarded because the time interval for image acquisition is less than two minutes. As just a small area of the image in Fig. 6(b) has the interference fringes, the region is extracted as a processing object. Six sets of interference fringe images are shown in Fig. 7, where eight spots with varying brightness, labeled 1–8, are selected. The light spots at these eight locations are utilized in the subsequent investigation to examine the algorithm's capacity to recover the interference fringes of light spots of various brightnesses.

For the experiments, 25 sampling sites with salt-and-pepper noise density values between 0.1 and 0.25 and at intervals of 0.01 were chosen. The interference fringe image acquired by CODES is double-type data with the salt noise point pixel value set to 2500.

### Comparative experimental analysis

In this study, six sets of 16-bit grayscale maps are used as experimental subjects and compared with ACmF^46^, DAMRmF^47^, IBLF^48^, MMAPF^49^, and TSF^50^. All comparison algorithms use open-source code, and both the proposed methods and the comparison algorithms exclusively employ the parameters suggested in the published articles. Metrics for evaluation included direct observation and RV offset. A brief description of each comparison algorithm is provided below:

ACmF: Replaces the standard median with the Cesáro average of the regular pixels to assign a new value to the window’s center pixel. Recursive as well, ACmF has a maximum window size of 11 × 11 and can employ higher filter window sizes if needed. It works slowly due to the recursive process.

DAMRmF: Adaptive median filters and pixel weight functions were employed in the adaptive conditions. In the Riesz mean, substitute a pixel weight function for the pixel similarity. High-density salt-and-pepper noise was subsequently eliminated by adaptive conditions employing adaptive median filtering and modified Riesz mean.

IBLF: The denoised images are obtained by generating intensity boundary-constrained images and reorganizing the generated boundary images. By extracting the lowest and higher extreme pixel values at each given place in the image, the boundary image maintains the edge data of the image. The rearrangement stage uses these divided boundary pictures to produce filtered images.

MMAPF: Impulse noise is removed using the change in the edge intensity values of the image and the difference in the edge intensity levels when using the minimum and maximum pools as the first layer. There are three programs within MMAPF. The first program is meant to enhance the less damaged images’ performance. Using min–max pools layered differently, the second program divides the image in half and uses them to collect image transitions. In the final step, more precise edge and boundary information are obtained by reorganization and an averaging pooling operation.

TSF: A two-stage filter is proposed to remove the high-density salt-and-pepper noise from an image. TSF includes two main stages: high-density noise reduction and low-density noise removal. Based on the median of the weak original pixel values, the first stage eliminates high-density noise; the second stage removes low-density noise based on the median of the maximum repeated pixel values. TSF does not require iteration and operates quickly. Nevertheless, because there are few original pixels in the case of high-density noise, the damaged pixel's value is not accurately reflected by the median value of the weak original pixel.

**Figure 8:**
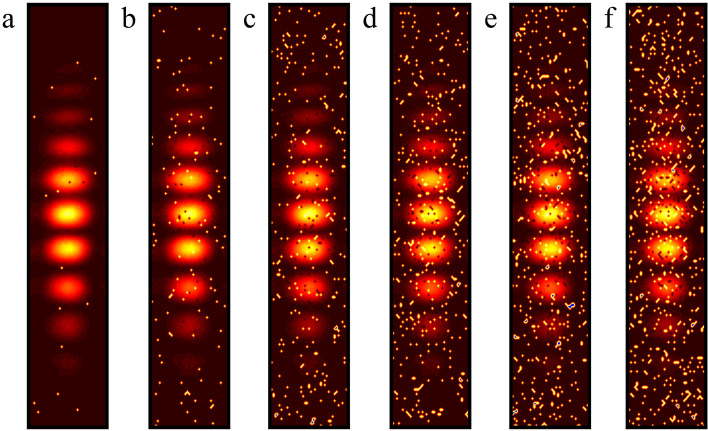
Interference fringe images with added salt-and-pepper noise. (**a**) noise density 0.01; (**b**) noise density 0.05; (**c**) noise density 0.10; (**d**) noise density 0.15; (**e**) noise density 0.20; (**f**) noise density 0.25.

**Figure 9:**
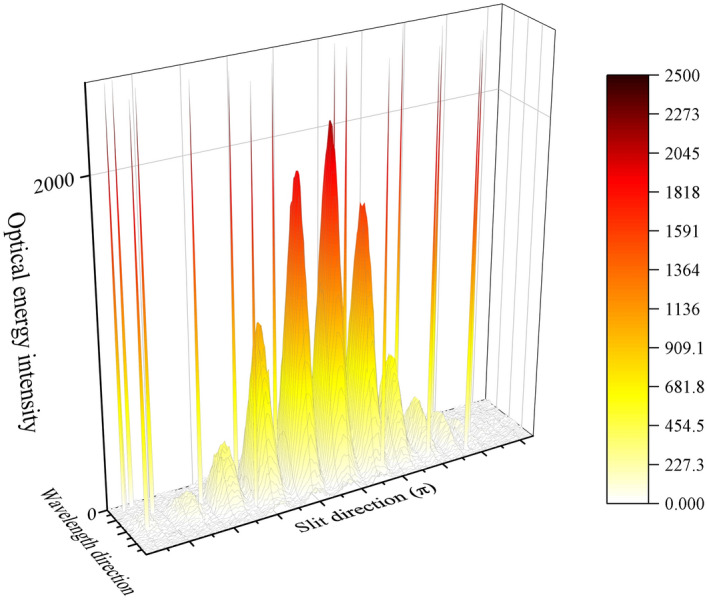
3D image of interference fringes with added salt-and-pepper noise. The noise density is 0.01.

**Figure 10:**
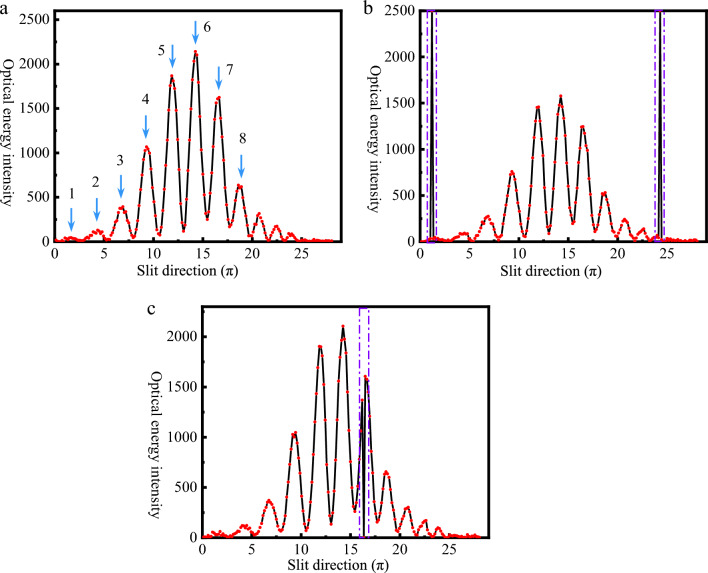
Interference fringes corresponding to three different wavelengths selected in Fig. 9. (**a**) 2D interference fringe curves without noise contamination; (**b**) 2D interference fringe curves in the presence of salt noise, which is in the purple dashed box; (**c**) 2D interference fringe curves in the presence of pepper noise, which is in the purple dashed box.

To study the denoising effect and fringe recovery of the algorithm under different conditions. In the experiment, 25 sampling points were selected from 0. 01 to 0.25 as the density value of salt-and-pepper noise, at intervals of 0.01. Fig 8 displays the interference fringe images with various densities of salt-and-pepper noise added. Fig 9 is a three-dimensional schematic of Fig. 8(a), which shows the interference fringe’s three-dimensional contour at a salt-and-pepper noise density of 0.01. Two-dimensional interference fringe curves at three different wavelengths selected from Fig. 9 demonstrate the differences between the interference fringes before and after salt-and-pepper noise contamination. Among them, Fig. 10(a) records the interference fringes’ 2D curves that aren't tainted by salt-and-pepper noise; Fig. 10(b)(c) shows the 2D curves of the interference fringes contaminated by pepper noise and salt noise, respectively. The eight peaks that run from left to right in Fig. 10(a) correspond to the eight interference fringe spots labeled in Fig. 7(a).

The mechanism through which salt-and-pepper noise results in RV errors in CODES is shown in Figs. 9,10. The change in the center wavelength of the absorption/emission lines of the stellar light caused by planetary perturbations is what causes the RV in the target star. The center wavelength of the absorption/emission line and the phase of the stellar light fringe are directly correlated: when the fringe's phase is altered by shifting the central wavelength, a signal representing radial velocity is produced. The two-dimensional interference fringes recorded by CCD store information in three dimensions: interference information along the slit, spectral information along the dispersion, and energy information shown by the bright and dark spots. The interference fringes’ profile is altered by the salt-and-pepper noise, which also modifies the energy information that the noisy image element records. Additionally, this alters the phase information that the interfering fringes carry, causing a mistake to be made in the computation of the interfering fringe phase variation Δφ, which ultimately leads to an inaccuracy in the RV inversion.

**Figure.11:**
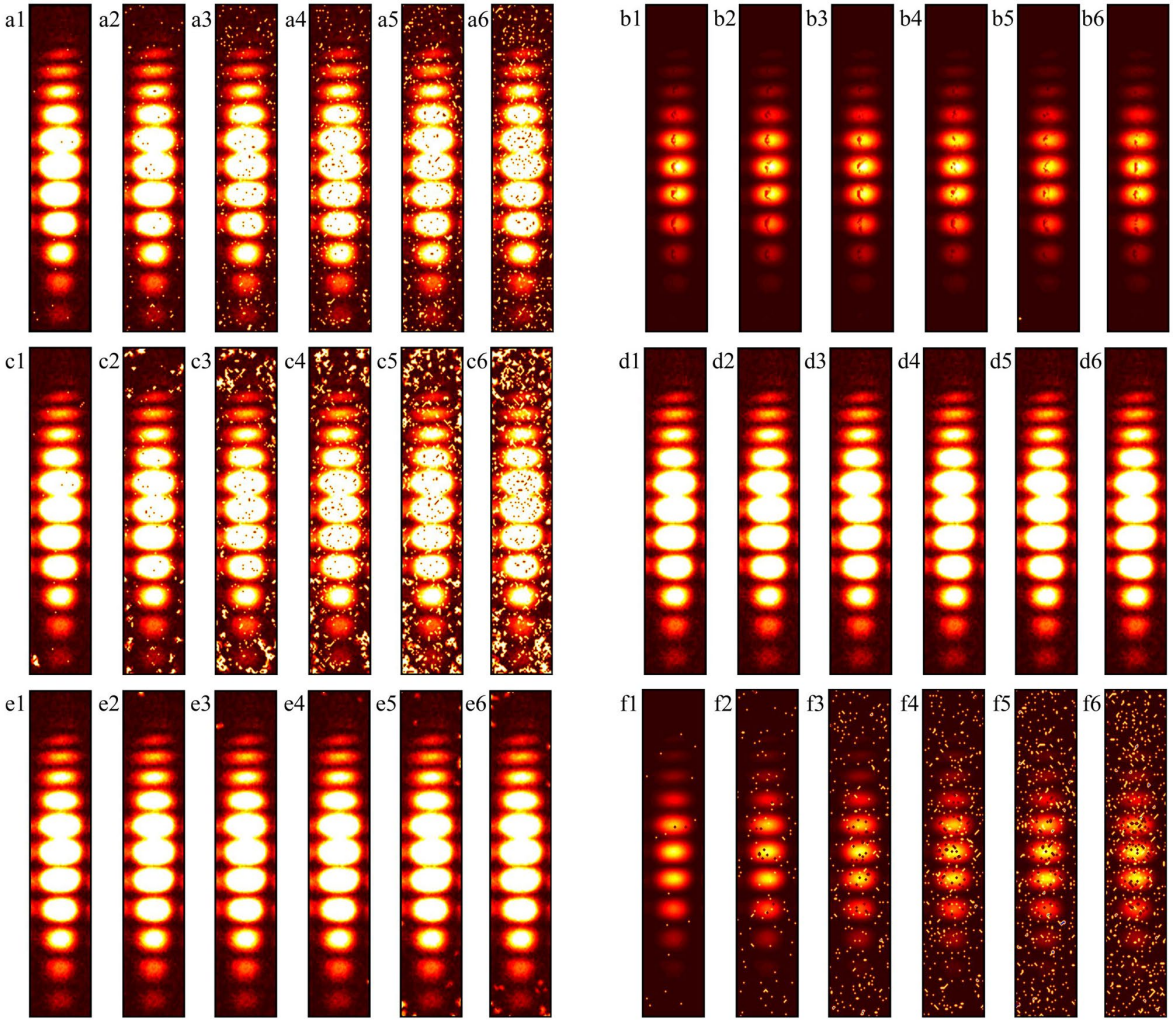
Interference fringe images processed by different algorithms. (**a**) ACmF; (**b**) CODESmF; (**c**) DAMRmF; (**d**) IBLF; (**e**) MMAPF; (**f**) TSF.

Figure 11 displays the interference fringes processed by the method developed in this study and the comparison algorithm, which shows six additional examples with noise densities of 0.01, 0.05, 0.10, 0.15, 0.20, and 0.25. First, a visual inspection of the denoised interference fringe image demonstrates that ACmF, DAMRmF, and TSF have poor peppercorn noise handling capabilities; even at low noise densities (e.g., 0.01), the peppercorn noise cannot be entirely eradicated. DAMRmF not only cannot effectively eliminate the pepper noise but even aggravates the noise; the interference fringe image processed by DAMRmF will have a greater area of salt noise, which is more severe the higher the noise density. Although both TSF and CODESmF are capable of recovering interference fringes more effectively, TSF performs worse than CODESmF at removing salt-and-pepper noise. CODESmF still has an excellent ability to cancel the salt-and-pepper noise, even though there is an uncanceled salt noise spot in Fig. 11 (b5); it can be concluded that this is an isolated case and the noise pixel does not alter the phase information of the interference fringes. To a limited extent, the interference fringe image is somewhat better recovered by CODESmF, however, the interference fringes that are recovered exhibit dark blotches in the center of the spots, indicating that it is still insufficient for recovering targets with high gray values. In conclusion, intuitive examination reveals that CODESmF has a strong capability to recover interference fringes and remove salt-and-pepper noise.

When the interference fringes’ bright and dark intensities differ, the denoising algorithm's results change. Eight different interference fringe spots are selected and the spot positions are shown in Fig. 7(a). The RV/phase offset between the interference fringe that is noise-contaminated and the interference fringe that has been subjected to the denoising procedure is the next step to take. When processing the interference fringes, the pixel points of the fringes are superimposed along the wavelength direction to prevent the chance of error brought on by the difference in the gray value of various fringe places. Eventually, an interference fringe curve representing this interference fringe image is obtained. A noise-free interference fringe image is processed using the aforementioned technique to produce an interference fringe curve I_1_; an interference fringe image with added salt-and-pepper noise is processed to obtain an interference fringe curve I_2_; and an interference fringe image after denoising is processed to obtain an interference fringe curve I_3_.

Cubic spline interpolation was used to fit the interference fringes I_1_, I_2_, and I_3_. The phase shift Δφ1 of the interference fringes generated by noise and the phase shift Δφ2 of the interference fringes after denoising out can be computed based on the tiny variations of the interference fringes in the same period. The phase offset’s direction is indicated by the positive and negative signs of the offset. The following equation can be used for calculating how much of a phase offset the denoising process has reduced.

22 $${\upeta }_{\text{off\_rec }}=1-\frac{\Delta {\varphi }_{1}-\Delta {\varphi }_{2}}{\Delta {\varphi }_{1}}$$

where η _off_rec_ reflects the ratio of phase error that the denoising method has fixed.

**Figure 12:**
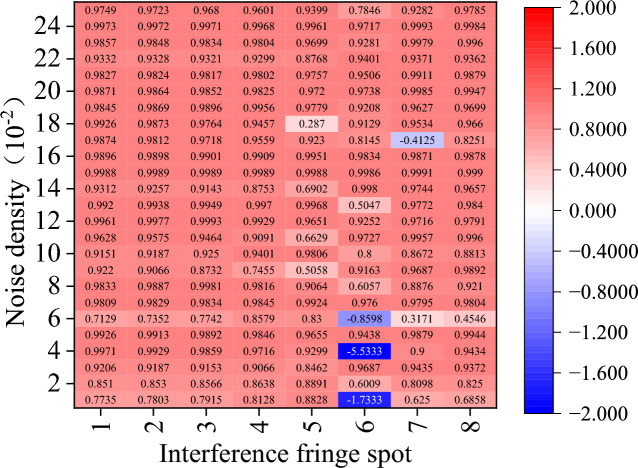
Amount of phase deviation that has been corrected after CODESmF denoising. Sequentially in the horizontal direction are the eight interference fringe spots shown in Fig. 7(a), and vertical direction is the added noise density sequentially 0.01–0.25 (the same as below).

**Figure 13:**
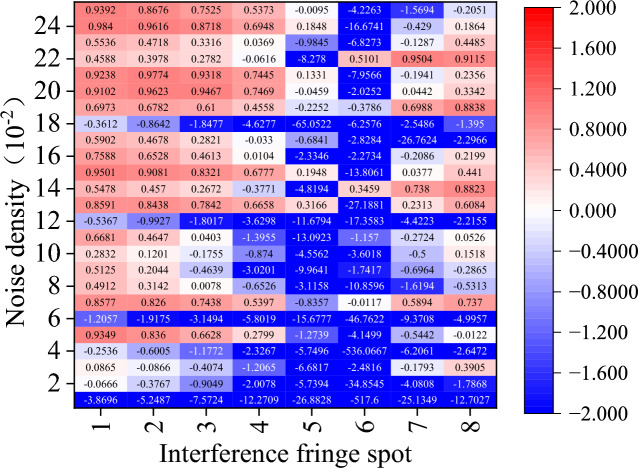
Amount of phase deviation that has been corrected after ACmF denoising.

**Figure 14:**
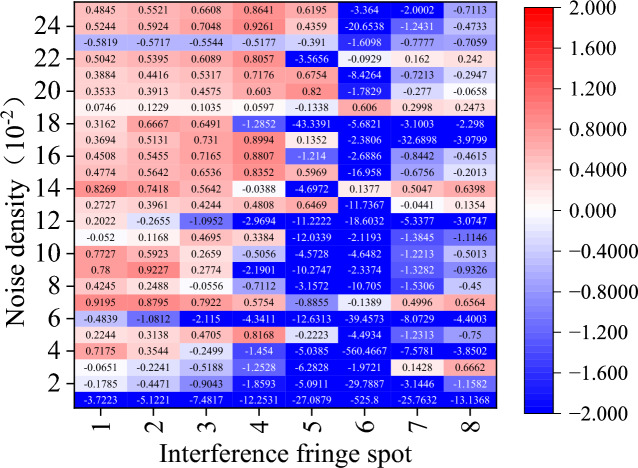
Amount of phase deviation that has been corrected after DAMRmF denoising.

**Figure 15:**
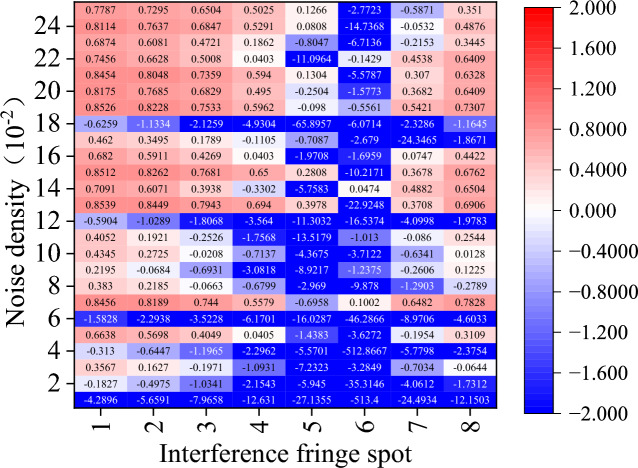
Amount of phase deviation that has been corrected after IBLF denoising.

**Figure 16:**
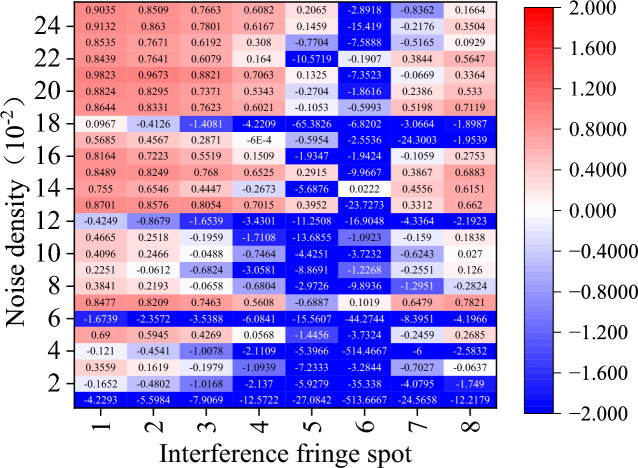
Amount of phase deviation that has been corrected after MMAPF denoising.

**Figure 17:**
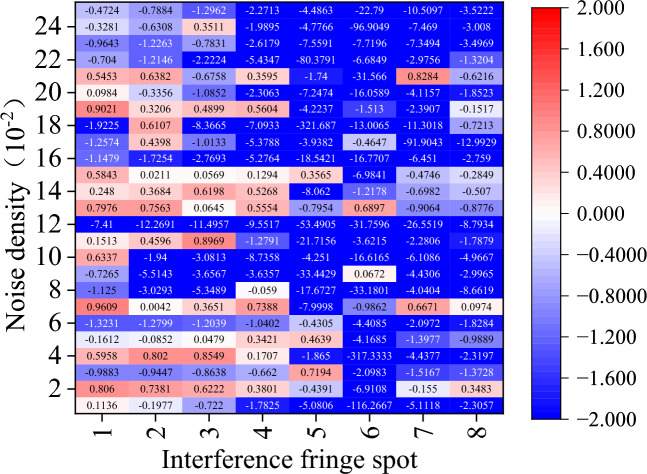
Amount of phase deviation that has been corrected after TSF denoising.

These six images, which range from Figs. 12, 13, 14, 15, 16 and 17 show the corrected phase errors as a percentage of the noise error for each algorithm for the eight spots of the first set interference fringe. A score close to 1 indicates a greater ability to recover the fringes. In this case, a negative number means that the algorithm’s capacity to recover the interference fringes is so inadequate that it not only fails to reduce the error but significantly worsens the interference with the fringes’ phase. The other comparison has a substantial number of recovery failures for the eight interference fringe spots carrying varying energies, and the probability of recovering the fringe failures increases with decreasing noise density.

Figure 12 shows that CODESmF performs exceptionally well in recovering interference fringes when their brightness is low. Regardless of the change in the salt-and-pepper noise density, CODESmF was able to reduce almost 70% of the phase error for the five low-brightness locations numbered 1–4 and numbered 8. (According to Eq. (21), there is a correlation between phase difference and RV; that is, more than 70% of the RV error could be removed.) Furthermore, CODESmF can remove over 90% of the phase error when the noise density is greater than 0.1, and in certain cases, up to 98%. The capacity of CODESmF for recovering the phase information of the interference fringe is reduced when the interference fringe is brighter. The presence of ${\upeta }_{\text{off\_rec}}$ being negative, observed at the three brightest interferometric fringe spots numbered 5–7, indicates that the CODESmF algorithm is at the moment unable to recover the interferometric fringes and interfering with the phase information that the interferometric fringes are carrying. Nevertheless, this phenomenon is more concentrated and happens less frequently; it mostly affects spot 6 which is exposed to low-dense salt-and-pepper noise. Moreover, compared to the other low-luminance areas, less phase error is eliminated by the CODESmF method for the three places numbered 5–7 when the salt-and-pepper noise density is the same. Naturally, for high-brightness interference fringe locations, the CODESmF algorithm can still remove over 90% of the phase error in most circumstances.

Regarding the other comparison methods, there is a significant rise in the cases where ${\upeta }_{\text{off\_rec}}$ is negative. In particular, the TSF algorithm is nearly impossible to employ for interference fringe recovery because it typically fails to recover interference fringes and, in the few instances that it does, typically only removes less than 60% of the phase error. While the performance of the other four comparison algorithms surpasses that of the TSF method, it is still far below that of the CODESmF algorithm. Interferometric fringe spots with higher energy or interferometric fringes with a low concentration of noise interference cannot be recovered by these four techniques. Numerous outcomes with negative values of ${\upeta }_{\text{off\_rec}}$ are obtained by these four recovery algorithms while working with the three locations numbered 5–7 or with noise densities less than 0.12.

At the same time, various algorithms share a few similar issues, issues on limited recovery for the three high-energy interference fringe spots, 5, 6, and 7, are particularly noteworthy. The interference fringes that the restoration algorithm processes still considerably differ from the original fringes and might even make the phase deviation worse. When processing the sixth spot with the highest energy, even the CODESmF algorithm, which has the best performance, revealed three instances of recovery failure; when the noise level was the same for all points, processing the sixth spot reduced the amount of phase deviation less than processing the other spots. In conjunction with the outcomes shown in Fig. 11b,a dark spot can be seen in the center of the interference fringe that has been processed by CODESmF. This black spot demonstrates that, at the peak position, the CODESmF algorithm’s recovery of the interference fringe's energy is still only slightly lower than the energy of the original fringe. This is because when the light intensity grows, the interference fringe spot's central region gathers an increasing amount of energy, and the correlation with nearby pixels weakens. This affects the algorithm that uses the correlation of nearby pixels.

The CODESmF method presented in this study is still superior to most of its competitors despite the presence of relatively few correction failures and a few sets of elimination phase deviations of less than 50%. The CODESmF method performs better for recovering interference fringes and removing salt-and-pepper noise. Even when the noise density and spot energy fluctuate, the algorithm still successfully eliminates more than 80% of the noise mistakes. The algorithm is particularly adapted to cope with high-density salt-and-pepper noise as the efficiency of salt-and-pepper noise error elimination gradually increases as the noise density grows. At the same time, there are a few places where recovery fails, although they are primarily found in a small number of the places with the most intense light energy. This is because when light energy rises, the core region of the spot receives an increasing proportion of the energy and the correlation with surrounding pixels weakens, which will affect the CODESmF algorithm, which is dependent on the correlation of nearby pixels.

Slight changes in the interfering fringes can cause mistakes in the RV computation since CODES derives the RV from the phase difference of the interfering fringes. Based on the intuitive evaluation as well as the objective comparison combined with the phase offset, the CODESmF algorithm does a good job of removing interference caused by salt-and-pepper noise on the phase of the interference fringes, recovering the interference fringes, and increasing the exoplanet RV inversion’s accuracy.

## Summary

This work describes the mechanism of salt-and-pepper noise’s impact on CODES, an exoplanet detection instrument for the asymmetric common optical path Sagnac interferometer, and designs a window-adaptive median filter denoising algorithm. Sub-extreme values are used to identify noisy pixel locations, which are then subjected to median filtering processes with a multilevel window. Phase error correction rate, denoising capability, and interference fringe recovery are employed to evaluate an algorithm’s performance. The experimental results demonstrate that the CODESmF algorithm can effectively eliminate the salt-and-pepper noise interference on the exoplanet RV inversion while also recovering the interference fringes effectively. The CODESmF method typically removes over 90% of phase/RV mistakes brought on by salt-and-pepper noise. It also has higher denoising performance and fringe restoration ability in the case of high-density salt-and-pepper noise contamination. Furthermore, the CODESmF algorithm performs better when dealing with low-energy interference fringes. When the noise level is more than 0.1 and the processing object is a low-luminance interference fringe, CODESmF removes about 98% of the phase inaccuracy. Eventually, the light intensity of the recovered spot is lower than that of the original spot (i.e., the high-brightness spot's central portion), and the phase error may be aggravated, which is the direction that needs to be improved in the next stage.

## Data Availability

The data that support the findings of this study are available from the corresponding author upon reasonable request.

## References

[1] Mayor, M. & Queloz, D. A Jupiter-mass companion to a solar-type star. *Nature* **378**, 355–359 (1995). 10.1038/378355a0

[2] Baycroft, T. A., Triaud, A. H. M. J. & Kervella, P. New evidence about HW Vir’s circumbinary planets from HipparcosGaia astrometry and a reanalysis of the eclipse timing variations using nested sampling. *Mon. Notices R. Astron. Soc.*10.1093/mnras/stad2794 (2023). 10.1093/mnras/stad2794

[3] Imtiaz, M., Hayat, T., Alsaedi, A. & Asghar, S. Slip flow by a variable thickness rotating disk subject to magnetohydrodynamics. *Results Phys.* **7**, 503–509. 10.1016/j.rinp.2016.12.021 (2017). 10.1016/j.rinp.2016.12.021

[4] Bischoff, R. *et al.* Young exoplanet transit initiative follow-up observations of the T Tauri star CVSO30 with transit-like dips. *Mon. Not. Royal Astron. Soc.* **511**, 3487–3500. 10.1093/mnras/stac293 (2022). 10.1093/mnras/stac293

[5] Koga, S. & Machida, M. N. Dust motion and possibility of dust growth in a growing circumstellar disk. *Mon. Not. Royal Astron. Soc.* **519**, 3595–3610. 10.1093/mnras/stac3503 (2022). 10.1093/mnras/stac3503

[6] Kumar, P., White, S. M., Stovall, K., Dowell, J. & Taylor, G. B. Pulsar observations at low frequencies: Applications to pulsar timing and solar wind models. *Mon. Not. Royal Astron. Soc.* **511**, 3937–3950. 10.1093/mnras/stac316 (2022). 10.1093/mnras/stac316

[7] Lewis, G. F. Gravitational microlensing time delays at high optical depth: Image parities and the temporal properties of fast radio bursts. *Mon. Not. Royal Astron. Soc.* **497**, 1583–1589. 10.1093/mnras/staa2044 (2020). 10.1093/mnras/staa2044

[8] Ge, J., Erskine, D. J. & Rushford, M. An externally dispersed interferometer for sensitive doppler extrasolar planet searches. *Publ. Astron. Soc. Pac.* **114**, 1016 (2002). 10.1086/342011

[9] van Eyken, J. C. et al. Results from upgrades to the radial velocity instrument, et, at the kpno 2.1 m. In *Ground-based Instrumentation for Astronomy*, vol. 5492, 445–451 (SPIE, 2004).

[10] van Eyken, J. C., Ge, J., Mahadevan, S. & DeWitt, C. First planet confirmation with a dispersed fixed-delay interferometer. *Astrophys. J.* **600**, L79 (2003). 10.1086/381574

[11] Wei, R. *et al.* Design and experimental test of a common-path coherent-dispersion spectrometer for exoplanet searches. *Publ. Astron. Soc. Pac.* **132**, 015003 (2019). 10.1088/1538-3873/ab503a

[12] Wu, Y. *et al.* Simulation and analysis of the coherent-dispersion spectrometer for exoplanet detection. *Mon. Not. Royal Astron. Soc.* **503**, 3032–3043 (2021). 10.1093/mnras/stab656

[13] Schubert, C. *et al.* Multi-loop atomic sagnac interferometry. *Sci. Rep.* **11**, 16121 (2021). 34373500 10.1038/s41598-021-95334-7 PMC8352864

[14] Kim, H., Kwon, O. & Moon, H. S. Pulsed sagnac source of polarization-entangled photon pairs in telecommunication band. *Sci. Rep.* **9**, 5031 (2019). 30903029 10.1038/s41598-019-41633-z PMC6430775

[15] Butler, R. P. *et al.* Attaining doppler precision of 3 m s-1. *Publ. Astron. Soc. Pac.* **108**, 500 (1996). 10.1086/133755

[16] Wang, J., Wan, X. & Jian, C. G. Development of monolithic michelson interferometer for rv measurement in ir. In Optical and Infrared Interferometry II, vol. 7734, 1309–1319 (SPIE, 2010).

[17] Erskine, D. & Ge, J. Novel interferometer spectrometer for sensitive stellar radial velocimetry. Tech. Rep., Lawrence Livermore National Lab.(LLNL), Livermore, CA (United States) (1999).

[18] Smolka, B., Kusnik, D. & Radlak, K. On the reduction of mixed gaussian and impulsive noise in heavily corrupted color images. *Sci. Rep.* **13**, 21035 (2023). 38030658 10.1038/s41598-023-48036-1 PMC10687184

[19] Yamaguchi, Y. *et al.* Edge-preserving smoothing filter using fast m-estimation method with an automatic determination algorithm for basic width. *Sci. Rep.* **13**, 5477 (2023). 37016031 10.1038/s41598-023-32013-9 PMC10073213

[20] Wu, Q., CHI, Y. B. & WANG, Z. Y. Effect of ccd noise on lossless compression of remote sensing images. *Opto-Electron. Eng.* **37**, 72–78 (2010).

[21] Thanh, D. N., Prasath, V. S. et al. Total variation l1 fidelity salt-and-pepper denoising with adaptive regularization parameter. In *2018 5th NAFOSTED Conference on Information and Computer Science (NICS)*, 400–405 (IEEE, 2018).

[22] Thanh, D. N. H. *et al.* Adaptive total variation l1 regularization for salt and pepper image denoising. *Optik* **208**, 163677 (2020). 10.1016/j.ijleo.2019.163677

[23] Mirza, M. S., Munaf, S. M., Azim, F., Ali, S. & Khan, S. J. Vision-based pakistani sign language recognition using bag-of-words and support vector machines. *Sci. Rep.* **12**, 21325 (2022). 36494382 10.1038/s41598-022-15864-6 PMC9734649

[24] Zhao, L. & Zhang, Z. A improved pooling method for convolutional neural networks. *Sci. Rep.* **14**, 1589 (2024). 38238357 10.1038/s41598-024-51258-6 PMC10796389

[25] Chen, J., Chen, J., Chao, H. & Yang, M. Image blind denoising with generative adversarial network based noise modeling. In *Proc.of the IEEE conference on computer vision and pattern recognition*, 3155–3164 (2018).

[26] Hwang, H. & Haddad, R. A. Adaptive median filters: New algorithms and results. *IEEE Trans. image Process.* **4**, 499–502 (1995). 18289998 10.1109/83.370679

[27] Wang, Z. & Zhang, D. Progressive switching median filter for the removal of impulse noise from highly corrupted images. *IEEE Transactions on Circuits Syst. II: Analog*. *Digit. Signal Process.* **46**, 78–80 (1999).

[28] Esakkirajan, S., Veerakumar, T., Subramanyam, A. N. & Premchand, C. Removal of high density salt and pepper noise through modified decision based unsymmetric trimmed median filter. *IEEE Signal Process. Lett.* **18**, 287–290 (2011). 10.1109/LSP.2011.2122333

[29] Erkan, U. & Gökrem, L. A new method based on pixel density in salt and pepper noise removal. *Turkish J. Electr. Eng. Comput. Sci.* **26**, 162–171 (2018). 10.3906/elk-1705-256

[30] Toh, K. K. V. & Isa, N. A. M. Noise adaptive fuzzy switching median filter for salt-and-pepper noise reduction. *IEEE Signal Process. Lett.* **17**, 281–284 (2009). 10.1109/LSP.2009.2038769

[31] Nodes, T. & Gallagher, N. Median filters: Some modifications and their properties. *IEEE Transactions on Acoust*. *Speech, Signal Process.* **30**, 739–746 (1982). 10.1109/TASSP.1982.1163951

[32] Shrestha, S. Image denoising using new adaptive based median filters. *arXiv preprint arXiv:1410.2175* (2014).

[33] Das, J., Das, B., Saikia, J. & Nirmala, S. Removal of salt and pepper noise using selective adaptive median filter. In *2016 International Conference on Accessibility to Digital World (ICADW)*, 203–206 (IEEE, 2016).

[34] Kunsoth, R. & Biswas, M. Modified decision based median filter for impulse noise removal. In *2016 International Conference on Wireless Communications, Signal Processing and Networking (WiSPNET)*, 1316–1319 (IEEE, 2016).

[35] Wan, F., Zhou, G. & Zhou, X. An adaptive fuzzy median filtering algorithm for salt and pepper noise removal. *J. Zhejiang Univ.* **46**, 445–453 (2019).

[36] Xu, G. An efficient switching median filter for the removal of salt and pepper noise. *J. Anhui Univ. Sci. Technol. Sci.* **37**, 33–39 (2017).

[37] Kamarujjaman, Maitra, M. & Chakraborty, S. A novel decision-based adaptive feedback median filter for high density impulse noise suppression. *Multimed. Tools Appl.* **80**, 299–321 (2021). 10.1007/s11042-020-09473-6

[38] Chan, R. H., Ho, C.-W. & Nikolova, M. Salt-and-pepper noise removal by median-type noise detectors and detail-preserving regularization. *IEEE Trans. image Process* **14**, 1479–1485 (2005). 16238054 10.1109/TIP.2005.852196

[39] Erskine, D. J. & Edelstein, J. High-resolution broadband spectral interferometry. In *Future EUV/UV and Visible Space Astrophysics Missions and Instrumentation* (eds Erskine, D. J. & Edelstein, J.) 158–169 (SPIE, 2003).

[40] Thanh, D. N., Prasath, V. S., Erkan, U. et al. An improved bpdf filter for high density salt and pepper denoising. In *2019 IEEE-RIVF International Conference on Computing and Communication Technologies (RIVF)*, 1–5 (IEEE, 2019).

[41] Sheik Fareed, S. B. & Khader, S. S. Fast adaptive and selective mean filter for the removal of high-density salt and pepper noise. *IET Image Process.* **12**, 1378–1387 (2018). 10.1049/iet-ipr.2017.0199

[42] Erkan, U., Enginoglu, S., Thanh, D. N. & Hieu, L. M. Adaptive frequency median filter for the salt and pepper denoising ˘problem. *IET Image Process.* **14**, 1291–1302 (2020). 10.1049/iet-ipr.2019.0398

[43] Xie, X., Shi, Z., Guo, W. & Yao, S. An adaptive image enhancement technique based on image characteristic. In *2009 2nd International Congress on Image and Signal Processing*, 1–5 (IEEE, 2009).

[44] Hernandez-Aguila, M., Olvera-Cervantes, J.-L., Perez-Ramos, A.-E. & Corona-Chavez, A. Methodology for the determination of human respiration rate by using doppler radar and empirical modal decomposition. *Sci. Reports* **12**, 8675 (2022). 10.1038/s41598-022-12726-z PMC9127072 35606407

[45] Pantke, D., Mueller, F., Reinartz, S., Kiessling, F. & Schulz, V. Flow velocity quantification by exploiting the principles of the doppler effect and magnetic particle imaging. *Sci. Rep.* **11**, 4529 (2021). 33633162 10.1038/s41598-021-83821-w PMC7907137

[46] Enginoglu, S., Erkan, U. & Memi¸s, S.,. Adaptive cesáro mean filter for salt-and-pepper noise removal. *El-Cezeri* **7**, 304–314 (2020).

[47] Memi, S. & Erkan, U. Different adaptive modified riesz mean filter for high-density salt-and-pepper noise removal in grayscale images. *Avrupa Bilim ve Teknoloji Dergisi* **23**, 359–367 (2021).

[48] Satti, P., Shrotriya, V., Garg, B. & Thanh, D. N. Intensity bound limit filter for high density impulse noise removal. *J. Ambient Intell. Humaniz. Comput.* **14**, 12453–12475 (2023). 10.1007/s12652-022-04328-4

[49] Satti, P., Sharma, N. & Garg, B. Min-max average pooling based filter for impulse noise removal. *IEEE Signal Process. Lett.* **27**, 1475–1479 (2020). 10.1109/LSP.2020.3016868

[50] Thanh, D. N., Hai, N. H., Prasath, V. S., Hieu, L. M. & Tavares, J. M. R. A two-stage filter for high density salt and pepper denoising. *Multimed. Tools Appl.* **79**, 21013–21035 (2020). 10.1007/s11042-020-08887-6

